# Cost-effectiveness of integrated disease management for high risk, exacerbation prone, patients with chronic obstructive pulmonary disease in a primary care setting

**DOI:** 10.1186/s12962-022-00377-w

**Published:** 2022-08-12

**Authors:** Andrew D. Scarffe, Christopher J. Licskai, Madonna Ferrone, Kevin Brand, Kednapa Thavorn, Doug Coyle

**Affiliations:** 1 Telfer School of Management, University of Ottawa, 55 Laurier Avenue East, Ottawa, ON K1N 6N5 Canada; 2 London Health Sciences Centre, Western University, London, ON Canada; 3 Lawson Health Research Institute, London, ON Canada; 4 Asthma Research Group Windsor Essex County Inc., ON Windsor, Canada; 5 Hotel-Dieu Grace Healthcare, Windsor, ON Canada; 6 Ottawa Hospital Research Institute, The Ottawa Hospital, Ottawa, ON Canada; 7 Faculty of Medicine, School of Epidemiology and Public Health, University of Ottawa, Ottawa, ON Canada; 8 Department of Clinical Sciences, College of Health and Life Sciences, Brunel University London, London, UK

**Keywords:** COPD, Chronic obstructive pulmonary disease, Cost-effectiveness, Cost-utility, Integrated disease management, Primary care

## Abstract

**Background:**

We evaluate the cost-effectiveness of the ‘Best Care’ integrated disease management (IDM) program for high risk, exacerbation prone, patients with chronic obstructive pulmonary disease (COPD) compared to usual care (UC) within a primary care setting from the perspective of a publicly funded health system (i.e., Ontario, Canada).

**Methods:**

We conducted a model-based, cost-utility analysis using a Markov model with expected values of costs and outcomes derived from a Monte-Carlo Simulation with 5000 replications. The target population included patients started in GOLD II with a starting age of 68 years in the trial-based analysis. Key input parameters were based on a randomized control trial of 143 patients (i.e., UC (n = 73) versus IDM program (n = 70)). Results were shown as incremental cost per quality-adjusted life year (QALY) gained.

**Results:**

The IDM program for high risk, exacerbation prone, patients is dominant in comparison with the UC group. After one year, the IDM program demonstrated cost savings and improved QALYs (i.e., UC was dominated by IDM) with a positive net-benefit of $5360 (95% CI: ($5175, $5546) based on a willingness to pay of $50,000 (CAN) per QALY.

**Conclusions:**

This study demonstrates that the IDM intervention for patients with COPD in a primary care setting is cost-effective in comparison to the standard of care. By demonstrating the cost-effectiveness of IDM, we confirm that investment in the delivery of evidence based best practices in primary care delivers better patient outcomes at a lower cost than UC.

**Supplementary Information:**

The online version contains supplementary material available at 10.1186/s12962-022-00377-w.

## Highlights

I. Interventions that can reduce the frequency and severity of exacerbations in patients who suffer from COPD have the potential to reduce the financial burden of COPD on the health system;
II. This is the first study that demonstrates the cost-effectiveness of integrated disease management for patients who suffer from COPD within a primary care environment;
III. This study makes the case for embedding Certified Respiratory Educators (CREs) within the primary care environment to improve the quality of life of patients who suffer from COPD, as well as alleviating unnecessary health services utilization and decreasing the overall financial burden of the disease on the health system.

**Supplementary Information:**

The online version contains supplementary material available at 10.1186/s12962-022-00377-w.

## Background

Globally, Chronic Obstructive Pulmonary Disease (COPD) is a leading cause of death and disability [1, 2]. In developed countries, COPD exacerbations are perceived to be one the largest economic burdens on the health system [3, 4]. Mittmann and colleagues (2008) highlight that 30–40 percent of the cost of caring for individuals with COPD is related to the management of exacerbations [5]. Moreover, COPD is recognized as the fourth leading cause of death after heart disease, cancer, and stroke [5].

Our study is based in Ontario, Canada. Ontario has a publicly funded healthcare system and is Canada’s most populated province (~ 14.7 million people), where it is estimated that 11.3 percent of adults between 55 and 64 years of age and 21.7 percent of adults that are 65 years of age and older suffer from physician-diagnosed COPD [6]. Relative to the general population, people with a diagnosis of COPD have a reduced quality of life, account for 24 percent of all hospitalizations and 24 percent of Emergency Department (ED) visits, and experience rates of hospitalization and ED visits that are 63 percent and 85 percent higher (respectively) than the general population [7]. In 2017, the government of Ontario reported that COPD exacerbations were responsible for more than 200,000 hospital inpatient days. To this end, therapeutic interventions that reduce the frequency of exacerbation events for a COPD patient have the potential to reduce the economic burden of COPD on Ontario’s health care system as well as health care systems globally [5, 8, 9].

COPD is a progressive lung disease characterized by worsening lung function, symptoms, quality of life, and increasing frequency and severity of exacerbations [10, 11]. To reflect the complexity of the disease, clinically and epidemiologically, COPD is characterized by the Global Initiative for Chronic Obstructive Pulmonary Disease (GOLD) in two ways. Clinically, the GOLD risk severity scale of ABCD reflects the phenotypical expression of COPD, based on symptom severity, quality of life, exacerbation frequency and severity. GOLD A and B subjects are at lower risk of exacerbation and GOLD C and D are at higher risk of exacerbation and of severe exacerbation requiring hospitalization. The clinical GOLD risk severity scale is highly relevant to both patients and health care professionals who deliver patient care. Epidemiologically, and for population modelling, the GOLD risk severity scale of I-IV is recommended. The I–IV stratification, with increasing severity, is based on the spirometry acquired lung function measurement of forced expiratory volume in one second (${FEV}_{1}$) [11, 12]. The GOLD scientific committee makes clear that, “spirometry remains key in the diagnosis, prognostication and the treatment of COPD with non-pharmacological therapies” [12]. In the GOLD I–IV severity scale, patients transition as their lung function deteriorates overtime until the patient dies or is classified as GOLD IV. Consistent with international guidelines, we have used GOLD A–D to describe our population in a clinically relevant manner and have used GOLD I–IV for population modelling.

As COPD progresses, patients experience an increase in the frequency and severity of COPD exacerbations. Once established, the frequent exacerbation phenotype (i.e., GOLD C and D) will persist over time in the absence of specific interventions to disrupt the pattern [13, 14]. That stated, international guidelines identify pharmacologic and nonpharmacologic best practices that, when implemented, substantially reduce the frequency of COPD exacerbations. However, the impact of these interventions across health systems is limited by the enduring challenge of inadequate guideline implementation. Proactive nonpharmacologic management strategies such as integrated disease management are highly effective, however they are frequently inadequately implemented. Because IDM programs are commonly inadequately implemented, patients with COPD (and by extension the health systems that care for them), do not realize the full clinical and health system benefits of these interventions. As a result of ineffective program implementation, COPD related exacerbations continue, thereby increasing patient morbidity and increasing financial cost to the health system. To improve the management of COPD within a primary care setting, the Primary Care Innovation Collaborative (PCIC) created the ‘Best Care’ integrated disease management (IDM) program to maximize the delivery of guideline-based, high impact, best practices for high risk, exacerbation prone patients with COPD [10].

The IDM program was initially evaluated by Ferrone and colleagues (2019) in a one year parallel group randomized controlled trial where IDM was demonstrated to improve lung function, health status, quality of life, and reduce severe COPD exacerbations, urgent physician and emergency department visits and hospitalization related to COPD exacerbations, compared to the usual standard of care [10]. The cost-effectiveness of the IDM program, however, remains unknown [10]. The intention of this study is to estimate the cost-effectiveness of the IDM program in comparison to the usual care (hereafter, UC) for high risk, exacerbation prone patients with COPD within a primary care setting.

## Methods

### Decision problem

The study is designed to address the decision problem facing decision makers within a publicly funded health care system in Canada (i.e., the province of Ontario), over whether they should fund the IDM program as an alternative program to UC. Thus, the study assesses the cost-effectiveness of the IDM program, relative to UC, for high risk, exacerbation prone, patients with COPD in a primary care setting.

### Type of economic evaluation

We conducted a within-trial and model-based cost-utility and cost-effectiveness analysis using a Markov model leveraging a Monte-Carlo simulation (MCS) technique with 5000 replications to predict long-term costs and clinical outcomes over a one-year (i.e., the trial-based analysis) and 30-year (i.e., the model-based analysis) time horizon. The benefit of using a MCS technique is it provides an unbiased estimate of outcomes in the presence of non-linear relationships between parameters and results.

### Perspective of the evaluation

Given the decision problem and the assumption that the objective of the health care system is to maximise population health while controlling financial cost, the analysis is from the perspective of a publicly funded health system.

### Treatments

#### ‘Best care’ integrated disease management (IDM)

The ‘Best Care’ integrated disease management program (IDM) intervention occurred in the primary care practice where patients normally received care [10]. The IDM intervention included proactive, standardized, guideline-based care including: “on-site spirometry [(i.e., within the primary care setting)], case management, education, and skills training, including self-management education by a certified respiratory educator (CRE) at baseline (1 h), 3 months post-enrollment (45 min), and either a telephone contact or in-person visit at 6 and 9 months (15–30 min)” [10]. Participants were also scheduled for an in-person visit at 12 months for the purposes of measuring study outcomes. The CREs who participated in the provision of care, “were all regulated healthcare professionals whose scope of practice included patient counseling and who have successfully completed a Canadian Network for Respiratory Care approved respiratory education program” [10]. As part of developing care management plans, the primary care physician was consulted at the end of every patient visit to approve of the patient care plan [10]. This consultation did not increase the cost of care (i.e., within the perspective of this evaluation) given care was provided within rostered family health teams (i.e., a blended remuneration model consisting of a base capitation payment, plus incentives and special payments).

#### Usual care (UC)

In Canada, patients with COPD typically receive care on an “as needed” or “needs to be assessed” basis by primary care practitioners [10, 15, 16]; care for patients in the usual care arm of the study was delivered according to normal practice patterns within the Family Health Teams [10]. Participants in the study attended scheduled study visits (i.e., no defined clinical intervention) on the same schedule as patients in the IDM group at 0, 3, 6, 9 and 12 months for the purposes of measuring study outcomes [10].

### Study subjects

Characteristics of our target population were consistent with a one-year multi-center randomized controlled trial that involved high risk, exacerbation prone, COPD patients in south-western Ontario (Canada). The analysis completed by Ferrone and colleages (2019) involved 74 participants in the UC group and 72 in the IDM group [10]. For the purposes of this analysis, participants who recorded a spirometry output that classified them as GOLD I at study initiation were removed because of low sample numbers (IDM, n = 2; UC, n = 1). This analysis is based on 73 participants in the UC group, and 70 participants in the IDM group. At study initiation, approximately 86 percent (n = 60) of the IDM group and 82 percent (n = 60) of the UC group were classified as GOLD D (Fisher’s Exact Test = p > 0.4). An analysis of baseline demographics suggest that the IDM and UC groups were well balanced in term of sex (${\chi }^{2}=1.65, df=1, p>0.05$), mean age (69.5 years vs. 67.7 years, respectively; ${t}_{140}=1.13, p>0.05$), and other demographic factors (see supplementary material for additional baseline demographic information).

The authors had full access to de-identified study data from the randomized controlled trial. All 143 participants included in the analysis had COPD Assessment Test (CAT) scores, spirometry testing, and health service utilization data completed for the first and final visit. See supplementary material for additional information.

### Model structure

**Fig. 1:**
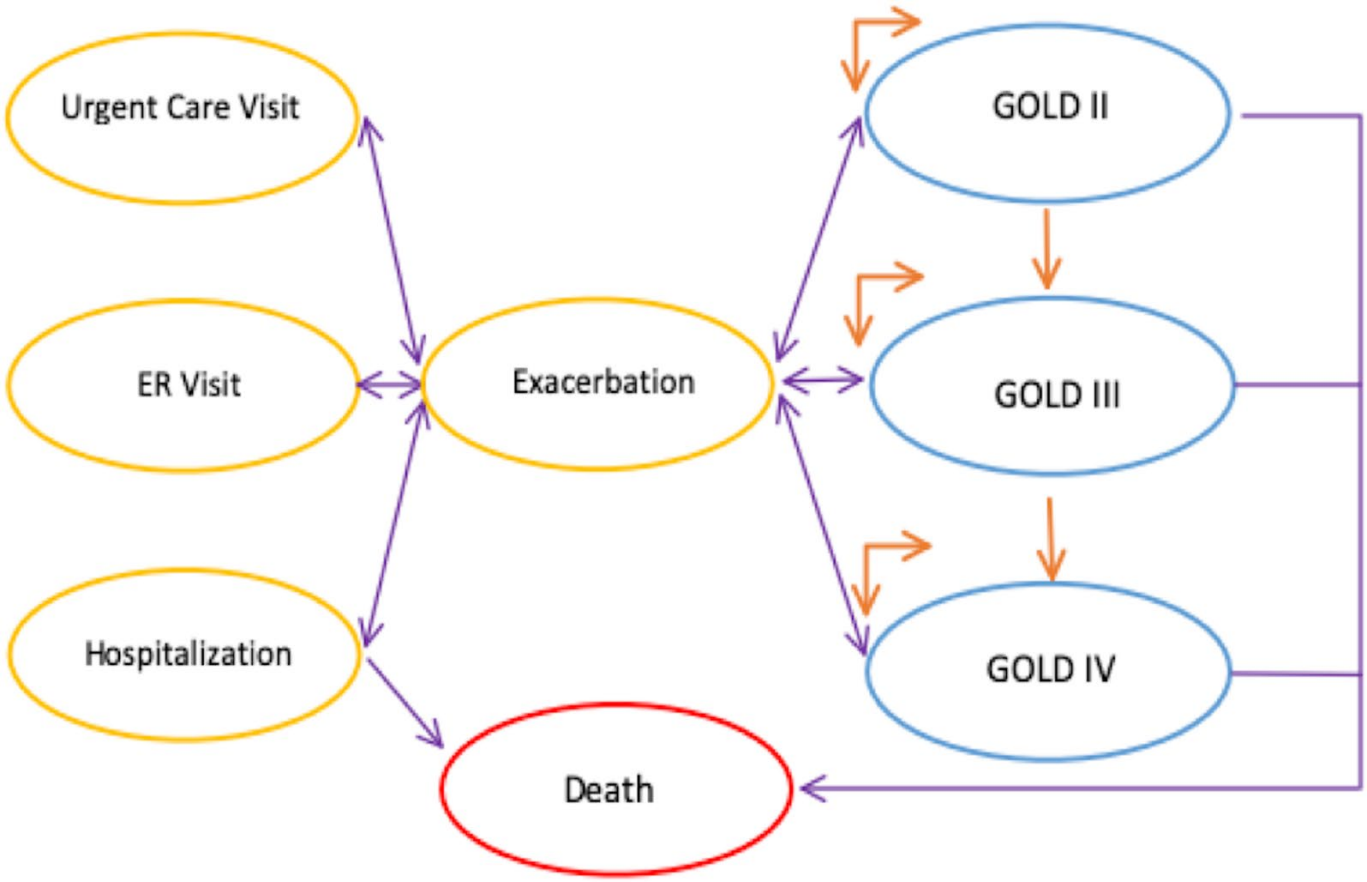
Schematic of Markov model. The orange cycling arrows reflect that an individual can remain within a health state at the end of an individual cycle (1 year). The unidirectional orange arrows reflect that an individual can transition to a worsening GOLD state in a non-recursive fashion (e.g., a patient cannot transition from GOLD III to II, but can transition from GOLD II to III) (i.e., P(Transition GOLD X to GOLD X + 1)). The unidirectional purple arrows reflect that an individual can die in any GOLD classification at the end of the cycle (i.e., P(Death | GOLD)), as well as die after experiencing an exacerbation and hospitalization (i.e., P(Death | Exacerbation & Hospitalization | GOLD)); death is an absorbing state. The purple bi-directional arrows between GOLD classification and Exacerbation reflect the P(Exacerbation | GOLD). The purple bi-directional arrows between Exacerbation and Urgent Care, ER Visit, and Hospitalization reflect the P(Specific Health Service Utilization | Exacerbation | GOLD).

A Markov model (see Fig. 1) underpins our simulation modeling. Markov modeling provides a method of modeling chronic conditions for which there is ongoing risk and multiple disease states (e.g., COPD) [17]. ${FEV}_{1}$ measurements are recognized as the most important parameter in the prediction of COPD related outcomes (e.g., mortality, hospitalization) at a population level [18]. We have therefore chosen to structure the COPD disease progression in our model based on ${FEV}_{1}$ measurements (i.e., GOLD I-IV) as determined by spirometry. The study focused on high risk, exacerbation prone patients with COPD and models patients in GOLD II-IV; GOLD I was excluded because of small sample size in the trial population (n = 2 IDM; n = 1 UC).

### Modeling lung function

COPD is a chronic disease where a patient’s health status worsens in a progressive, non-reversible, fashion [3, 11, 19, 20]. The model assumes that a patient cannot transition beyond the next progressive disease state (or death) in a single cycle (i.e., a patient must either remain in their current state, transition to the next state, or die, in a mutually exclusive and exhaustive fashion within each cycle) (see Fig. 1); death is an absorbing state [21]. Because the IDM program was not designed to improve airway functioning, but rather improve the management of patients with COPD, the model assumes that the annual probabilities of transition (i.e., disease progression) between GOLD II and III and GOLD III and IV, respectively, are the same across the UC group and IDM group (see Table 1); a beta distribution was assigned to these probabilities.

Lung function measured as post-bronchodilator ${FEV}_{1}$ was completed to American Thoracic Society and European Thoracic Society (ATS/ERS) standards at study initiation and patients were classified as GOLD I-IV [10]. The lung function measured by airflow limitation for patients with COPD are by definition not fully reversible and lung function declines over time [3]. The GOLD scientific committee recommends severity classification at time of first assessment [3]. Given this recommendation, our analysis was based on GOLD stratification defined at the initial visit.

Each GOLD state has its’ own conditional probability of exacerbation, as well as conditional probability of an urgent care visit, emergency room (ER) visit, and hospitalization, which are unique to the UC and IDM groups. Age dependent probabilities of death are informed by the age stratified all-cause probability of death for Ontario (2015–2017) in combination with the relative risk of mortality for each GOLD state [22, 23]. It is assumed that the only state which increases an individual’s probability of death (i.e., relative to the other patients within the model), are those patients who experience an exacerbation and are hospitalized. Probabilities of death for patients who have an exacerbation and are hospitalized are informed by the age dependent probability of death, the age stratified Ontario mortality rate (2017), and the hazard ratio for hospitalized mortality for each GOLD state (see Table 1) [22–24]. The model assumed that the IDM program did not have a direct effect on the probability of death, but rather had an indirect effect through the reduction in the probability of exacerbation and subsequently hospitalization (i.e., the probabilities of death were the same for the UC and IDM group and stratified by age and GOLD classification).

**Table 1:** Model Parameters

Parameters	Base Estimate	Probability Distribution*	References
Discount Rate	1.5%	Fixed	[28]
Willingness to Pay Value	$50,000	Fixed	
Relative Risk of Mortality- GOLD II	1.44	Log normal (1.135, 1.778)	[22]
Relative Risk of Mortality- GOLD III	2.04	Log normal (1.495, 2.569)	[22]
Relative Risk of Mortality- GOLD IV	4.24	Log normal (1.496, 3.921)	[22]
Death Hazard Ratio (Hospitalization)- GOLD II	1.5	Log normal (1.260, 1.757)	[53]
Death Hazard Ratio (Hospitalization)- GOLD III & GOLD IV	2.7	Log normal (2.066, 3.214)	[53]
Utility values			
EQ-5D- GOLD II- UC	0.711	Resample (min = 0.377, max = 0.922, median = 0.733)	[10]
EQ-5D- GOLD III- UC	0.687	Resample (min = 0.472, max = 0.959, median = 0.673)	[10]
EQ-5D- GOLD IV- UC	0.708	Resample (min = 0.544, max = 0.889, median = 0.726)	[10]
EQ-5D- GOLD II- IDM	0.817	Resample (min = 0.566, max = 0.978, median = 0.836)	[10]
EQ-5D- GOLD III – IDM	0.798	Resample (min = 0.548, max = 0.978, median = 0.759)	[10]
EQ-5D- GOLD IV- IDM	0.720	Resample (min = 0.540, max = 0.801, median = 0.771)	[10]
Death/ Dead- UC & IDM	0	Fixed	
Annual transition probabilities			
Transition from GOLD II to GOLD III	0.08	Beta (7, 84)	[10]
Transition from GOLD III to GOLD IV	0.05	Beta (2, 39)	[10]
Usual care- per annual cycle			
Probability of Exacerbation- GOLD II	0.86	Beta (38, 6)	[10]
Probability of Exacerbation- GOLD III	0.73	Beta (16, 6)	[10]
Probability of Exacerbation- GOLD IV	0.86	Beta (6, 1)	[10]
Probability of Urgent Care Visit GOLD II	0.89	Beta (34, 4)	[10]
Probability of Urgent Care Visit GOLD III	0.75	Beta (12, 4)	[10]
Probability of Urgent Care Visit GOLD IV	0.98	Beta (6, 0.1)	[10]
Probability of ER Visit- GOLD II	0.45	Beta (17, 21)	[10]
Probability of ER Visit- GOLD III	0.50	Beta (8, 8)	[10]
Probability of ER Visit- GOLD IV	0.50	Beta (3, 3)	[10]
Probability of Hospitalization- GOLD II	0.18	Beta (7, 31)	[10]
Probability of Hospitalization- GOLD III	0.25	Beta (4, 12)	[10]
Probability of Hospitalization- GOLD IV	0.33	Beta (2, 4)	[10]
# of Urgent Care Visits- GOLD II	2.684	Log normal (2.074, 3.185)	[10]
# of Urgent Care Visits- GOLD III	2.124	Log normal (1.178, 2.964)	[10]
# of Urgent Care Visits- GOLD IV	3.833	Log normal (0.603, 2.628)	[10]
# of ER Visits- GOLD II	0.474	Log normal (0.326, 0.683)	[10]
# of ER Visits- GOLD III	0.75	Log normal (0.403, 1.322)	[10]
# of ER Visits- GOLD IV	0.833	Log normal (0.262, 2.112)	[10]
# of Hospitalizations- GOLD II	0.263	Log normal (0.123, 0.554)	[10]
# of Hospitalizations- GOLD III	0.438	Log normal (0.166, 1.095)	[10]
# of Hospitalizations- GOLD IV	0.333	Log normal (0.105, 1.016)	[10]
IDM—per annual cycle			
Probability of Exacerbation- GOLD II	0.26	Beta (12, 35)	[10]
Probability of Exacerbation- GOLD III	0.47	Beta (9, 10)	[10]
Probability of Exacerbation- GOLD IV	0.50	Beta (2, 2)	[10]
Probability of Urgent Care Visit GOLD II	0.75	Beta (9, 3)	[10]
Probability of Urgent Care Visit GOLD III	0.44	Beta (4, 5)	[10]
Probability of Urgent Care Visit GOLD IV	0.95	Beta (2, 0.1)	[10]
Probability of ER Visit- GOLD II	0.42	Beta (5, 7)	[10]
Probability of ER Visit- GOLD III	0.11	Beta (1, 8)	[10]
Probability of ER Visit- GOLD IV	0.50	Beta (1, 1)	[10]
Probability of Hospitalization- GOLD II	0.17	Beta (2, 10)	[10]
Probability of Hospitalization- GOLD III	0.22	Beta (2, 7)	[10]
Probability of Hospitalization- GOLD IV	0.50	Beta (1, 1)	[10]
# of Urgent Care Visits- GOLD II	1.667	Log normal (1.069, 2.324)	[10]
# of Urgent Care Visits- GOLD III	0.667	Log normal (0.250, 1.592)	[10]
# of Urgent Care Visits- GOLD IV	1.5	Log normal (0.701, 2.501)	[10]
# of ER Visits- GOLD II	0.417	Log normal (0.209, 0.812)	[10]
# of ER Visits- GOLD III	0.222	Log normal (0.042, 1.108)	[10]
# of ER Visits- GOLD IV	0.5	Log normal (0.086, 2.256)	[10]
# of Hospitalizations- GOLD II	0.417	Log normal (0.098, 1.577)	[10]
# of Hospitalizations- GOLD III	0.222	Log normal (0.068, 0.716)	[10]
# of Hospitalizations- GOLD IV	0.5	Log normal (0.086, 2.256)	[10]
Health service costs per visit			
Urgent care- outpatient Physician visits	$75.93	Gamma (10,892.910, 0.00697)	[5]
Urgent care- laboratory & diagnostic tests	$19.62	Gamma (107.360, 0.186)	[5]
Urgent care- transportation	$28.63	Gamma (61.975, 0.462)	[5]
Urgent care- medication charges	$32.36	Gamma (673.687, 0.048)	[5]
ER- ER visit	$313.38	Fixed	[5]
ER- transportation	$299.98	Gamma (40.930, 7.329)	[5]
ER- medication changes	$26.14	Gamma (80.414, 0.325)	[5]
Hospitalization- hospital stay	$8,787.93	Gamma (63.791, 137.760)	[5]
Hospitalization- laboratory & diagnostic tests	$1,845.86	Gamma (309.379, 5.966)	[5]
Hospitalization- transportation	$155.59	Gamma (24.237, 6.420)	[5]
Death	$0	Fixed	
Annual treatment cost			
Medical program director	$46.67	Gamma (25, 1.867)	[10]
Program coordinator	$80.00	Gamma (25, 3.2)	[10]
Certified respiratory educator	$216.00	Gamma (25, 8.64)	[10]
Spirometry	$10.00	Gamma (25, 0.4)	[10]
Computer	$2.40	Gamma (25, 0.096)	[10]
Spirometry filters	$5.25	Fixed	[10]

^*^All estimates are calculated to be on a per-year basis. Beta distributions are specified by alpha and beta. Log normal distributions are specified by lower and upper limits of the 95% confidence intervals. Gamma distributions are specified by shape and scale parameters

### Time horizon

The trial-based analysis adopted a time-horizon of one year starting at the age of 68 (i.e., the median age in our UC group). In the model-based analysis, we adopted a 30-year time horizon with a starting age of 60 years of age (i.e., the “Base Case” scenario). The model-based analyses used a cycle length of 1 year, which is consistent with how other published studies have modelled COPD progression [19, 25]. The starting age of 60 in our model-based analysis differs from the starting age of 68 in our trial-based analysis in an effort to generalize our findings. We did run a scenario analysis of a 20-year time horizon starting at 68 years of age to remain consistent with the starting age in our trial-based analysis and noted that results remained consistent with the trial-based and “Base Case” analyses (see Scenario Analysis for additional information). The effects of the IDM program were assumed to be sustained beyond the initial year. The assumption that the treatment effect is sustained beyond the trial is consistent with the findings of similar IDM programs for moderate-to-severe COPD patients that have demonstrated that treatment effects of self-management programs continue to reduce urgent physician and emergency room visits as well as hospitalizations [26]. Because the nature of this analysis was focused on the incremental benefit of IDM versus UC, half-cycle correction was not applied as it was expected to have a minimal effect on the results [27]. Cost and outcomes were discounted at a rate of 1.5% per period (i.e., per annum) as per the Canadian Agency for Drugs and Technologies in Health’s (CADTH) recommendation [28]; a scenario analysis was conducted discounting future events at a rate of 5%.

### Health utility values

Utility values were derived from the original trial data provided by Ferrone and colleagues (2019). CAT scores from Ferrone and colleagues (2019) were converted to EQ-5D estimates using an algorithm for converting CAT scores to EQ-5D estimates of utility for the purposes of health economic evaluations [29]. The regression equation specifically interprets the CAT scores that relate to measures of: chest tightness, activity, confidence, and energy. These specific CAT measures have been found to be statistically significant in the estimation of EQ-5D [29]. Utility values were conditional to a patient’s GOLD classification at the end of the cycle as well as if the patient belonged to the UC or IDM group. QALY was calculated as the aggregate sum of utility across the time horizon. Our assumption that there is a difference in utility between IDM and UC groups is supported by the findings of Ferrone and colleagues (2019) who report a statistically significant difference in CAT scores between the UC group and the IDM group upon trial completion [10].

To incorporate uncertainty in utility estimates into the model, we leveraged a resampling with replacement strategy of utility estimates that were dependent on treatment group (i.e., IDM vs. UC) and GOLD classification. Resampling with replacement was perceived to be a favourable strategy, relative to imposing a theoretical distribution, because of a small sample size in GOLD IV for UC and IDM groups, the use of estimation through linear regression, and a high degree of covariance between individual measures within the CAT score. Resampling with replacement within MCS is recognized as a suitable method when theoretical distributions are unjustified [30, 31].

### Costs

Costs were adjusted to 2019 Canadian dollars using the Bank of Canada Inflation Calculator [32]. Intervention cost estimates were obtained directly from the Asthma Research Group Windsor-Essex County Inc. (ARGI) based on actual program costs. For the costs related to the Medical Program Director (MPD) and the Program Coordinator (PC), base estimates assumed that the cost of these positions would be divided across 1,500 patients (i.e., the estimated number of patients a MPD/ PC could reasonably manage on their roster). For the costs related to the CRE (i.e., salary, spirometry device, and personal computers) it was assumed in the base case that the cost of each CRE would be divisible by 500 patients (i.e., the estimated number of patients a CRE could reasonably manage on their roster); in addition to the human resource costs of delivering care, it was also assumed that the cost of physical equipment (e.g., spirometry device, personal computers) would be replaced annually. Because we did not have explicit information on the cost variation, we chose a coefficient of variation of 20% for each cost estimate as a proxy of uncertainty in the base estimate; except for spirometry filters (i.e., a fixed cost). Average health system costs (e.g., urgent care, emergency room visits, hospitalizations) were derived from the literature and adjusted for inflation [5]. All health system cost(s) were modeled using a gamma distribution.

### Software and uncertainty analysis

The MCS model was built in Microsoft Excel (version 16.32) and data analysis was completed in R (version 1.2.5033) with the assistance from the “BCEA”, “dplyr”, “epitools”, and “ggplot2” packages. MCS was adopted to derive the expected values for costs and utilities by sampling inputs from the uncertainty distributions as described in Table 1. For the MCS, probability distributions related to: relative risks, hazard ratios, exacerbations, costs (i.e., both health system and intervention), utilities, and the probability and frequency of health service utilization were incorporated into the analysis. In this study, estimates of incremental costs and incremental QALYs were obtained by running 5,000 replications of the MCS model; each replication employed values from their corresponding probability distributions. The probability of health service utilization and disease progresion were modelled using beta distributions; relative risks, hazard ratios and frequency of health service utilization were modelled using log normal distributions; health system costs were modelled using gamma distributions (see MERGEFORMAT Table 1). We also estimated the price that policymakers would be willing to pay to gain access to remove the possibility of making the wrong decision (i.e., Per Patient Expected Value of Perfect Information (ppEVPI)) using the Bayesian cost-effectiveness analysis (“BCEA”) package in R. Extensive uncertainty analyses was undertaken on the input parameters using both linear and logistic models to explore the ppEVPI in greater detail. Specifically, we determined which of the input parameters were statistically significant in determining if IDM was cost-effective and then estimated the Expected Value of Perfect Partial Information (EVPPI) of different input parameters to determine which parameters were most informative to the estimation of cost-effectiveness (see supplementary material for additional information).

## Results

IDM was found to significantly increase QALYs (95% CI: (0.098, 0.106)) and reduce cost (95% CI: (-$257, -$277)) in comparison to UC within a time horizon of one year (i.e., it was dominant relative to UC) (see Table 2). As expected, IDM did not have a significant effect on expected life years when simulated for one year in comparison to UC. The model-based analyses provided consistent results, suggesting that IDM improved both QALYs and life expectancy compared to UC. In our model-based analyses, our “Base Case” scenario extends the time horizon to 30-years starting at 60 years of age. When comparing the IDM versus UC groups in the “Base Case”, we report a mean increase of 1.732 QALYs per person (95% CI: (1.685, 1.779)) in favour of the IDM group (see Table 3). On average, the IDM group cost significantly less per person (95% CI: (-$3748, -$4198)) than the cost per person of UC. We also observed that life expectancy (i.e., life years) was significantly increased for the IDM group relative to the UC group in the “Base Case” scenario (95% CI: (0.232, 0.257)). Additionally, the IDM group demonstrated significantly fewer exacerbations than the UC group [10]. The IDM group had less health service utilization/ expenditure as a result of fewer exacerbations, and reported an overall improvement in quality of life (QALY) in comparison to UC [10].

**Table 2:** Trial-based results (i.e., 1 year simulation starting at age 68)

Probabilistic Results	Life Years	Costs	QALYs	ICUR	ICER	Net Benefit
Usual Care Group	0.97	$921	0.686			
IDM Group	0.97	$654	0.788			
Incremental	0.00	− $267*	0.102*	Dominant	Dominant	$ 5,360

Dominance/ Dominant = a treatment that is less costly and results in improved health outcomes; ICER = Incremental Cost Effectiveness Ratio; ICUR = Incremental Cost Utility Ratio; Net Benefit = Differential in QALY * Willingness to Pay per QALY—Differential in Cost; QALYs = Incremental Quality Adjusted Life Years (IDM vs. UC)

^*^Statistically significant to p < 0.01

**Table 3:** Base-Case Results (i.e., 30-year simulation starting at age 60)

Probabilistic results	Life years	Costs	QALYs	ICUR	ICER	Net benefit
Usual care group	16.011	$18,100	11.233			
* GOLD II*		*$7,588*	*5.885*			
* GOLD III*		*$7,370*	*3.946*			
* GOLD IV*		*$3,152*	*1.403*			
IDM group	16.255	$14,137	12.965			
* GOLD II*		*$5,632*	*6.854*			
* GOLD III*		*$4,040*	*4.651*			
* GOLD IV*		*$4,465*	*1.460*			
Incremental	0.244*	-$3,973*	1.732*	Dominant	Dominant	$ 90,576

Dominance/ Dominant = a treatment that is less costly and results in improved health outcomes; ICER = Incremental Cost Effectiveness Ratio; ICUR = Incremental Cost Utility Ratio; Net Benefit = Differential in QALY * Willingness to Pay per QALY – Differential in Cost; QALYs = Incremental Quality Adjusted Life Years (IDM vs. UC)

^*^Statistically significant to p < 0.001

**Fig. 2:**
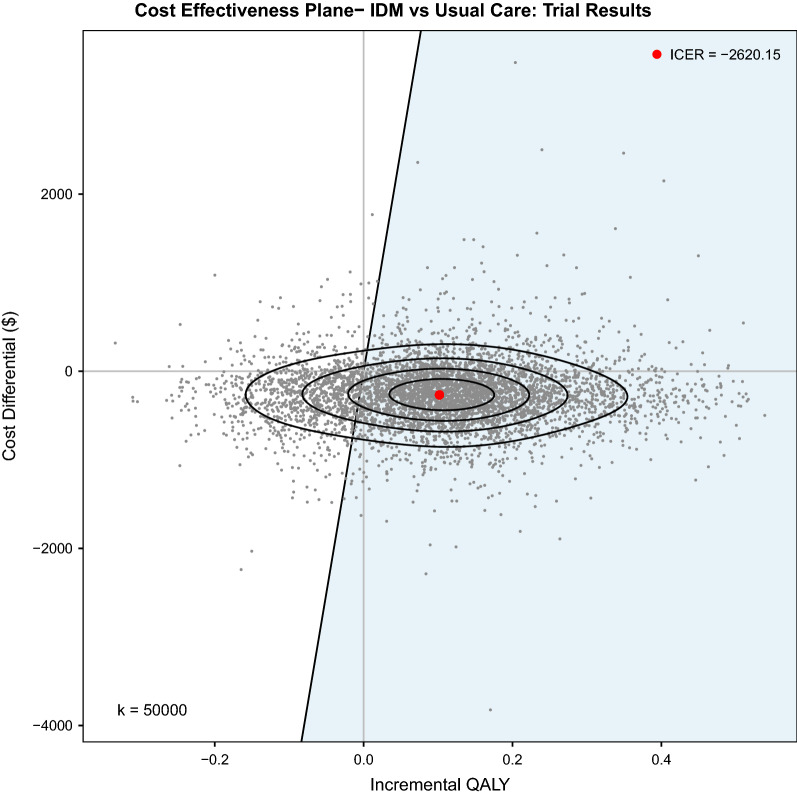
Cost Effectiveness Plane: Trial-Based Results. This graph plots the MCS results of 5,000 replications of IDM vs. UC for the trial-based results and plots the cost effectiveness plane against a willingness to pay (WTP) threshold of $50,000 (CAN) per QALY (k = 50,000). The ICER (Incremental Cost-Effectiveness Ratio) is akin to the ICUR (Incremental Cost-Utility Ratio) given incremental cost of IDM is plotted against incremental QALY; the ICER reflects that IDM is dominant to UC. The blue shaded area reflects the sustainability area (i.e., below the WTP threshold; P(Sustainability Area) = 0.7878). The ellipses divide the observed bivariate distribution of the outcomes (i.e., $$\mathrm{\Delta QALY},\mathrm{ \Delta Cost})$$ with an estimated probability density function of a constant value. Given five areas (i.e., four ellipses), each area reflects 20% of outcomes.

**Fig. 3:**
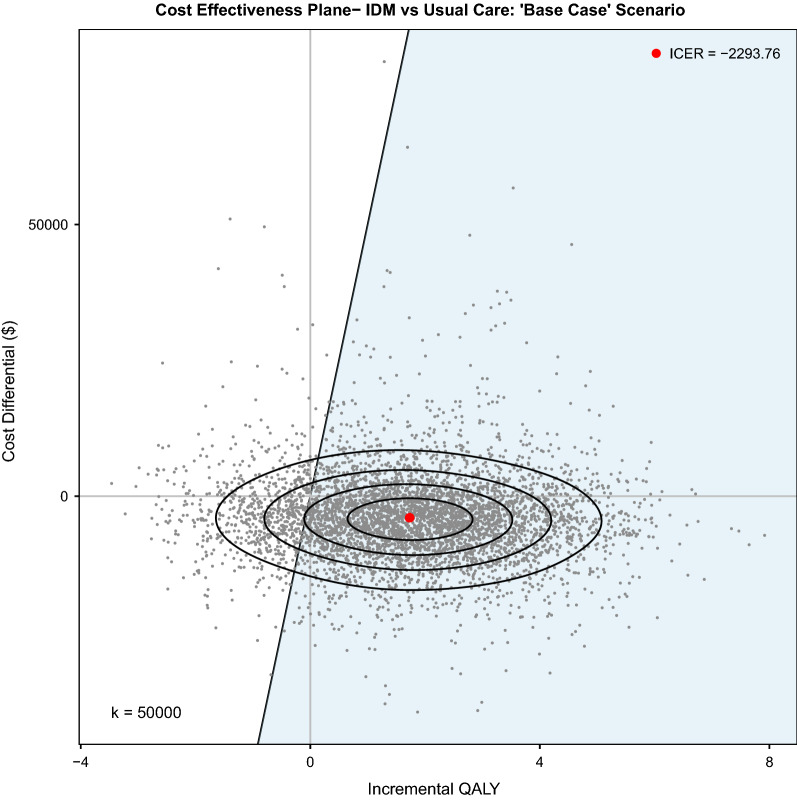
Cost Effectiveness Plane: “Base Case” Scenario. This graph plots the MCS results of 5,000 replications of IDM vs. UC in the “Base Case” scenario and plots the cost effectiveness plane against a willingness to pay (WTP) threshold of $50,000 (CAN) per QALY (k = 50,000). The ICER (Incremental Cost-Effectiveness Ratio) is akin to the ICUR (Incremental Cost-Utility Ratio) given incremental cost of IDM is plotted against incremental QALY; the ICER reflects that IDM is dominant to UC. The blue shaded area reflects the sustainability area (i.e., below the WTP threshold; P(Sustainability Area) = 0.8530). The ellipses divide the observed bivariate distribution of the outcomes (i.e., $$\mathrm{\Delta QALY},\mathrm{ \Delta Cost})$$ with an estimated probability density function of a constant value. Given five areas (i.e., four ellipses), each area reflects 20% of outcomes.

Analysis demonstrates that IDM was dominant compared to UC (see Fig. 2 and Fig. 3). The trial-based results yield an incremental net-benefit of $5,360 (95% CI: ($5175, $5546)) assuming a willingness to pay threshold (WTP) of $50,000 per QALY. Comparatively against a longer time horizon, the “Base Case” scenario yields a net-benefit of $90,576 (95% CI: ($88211, $92941)) assuming a WTP of $50,000 per QALY.

### Uncertainty analysis

**Fig. 4:**
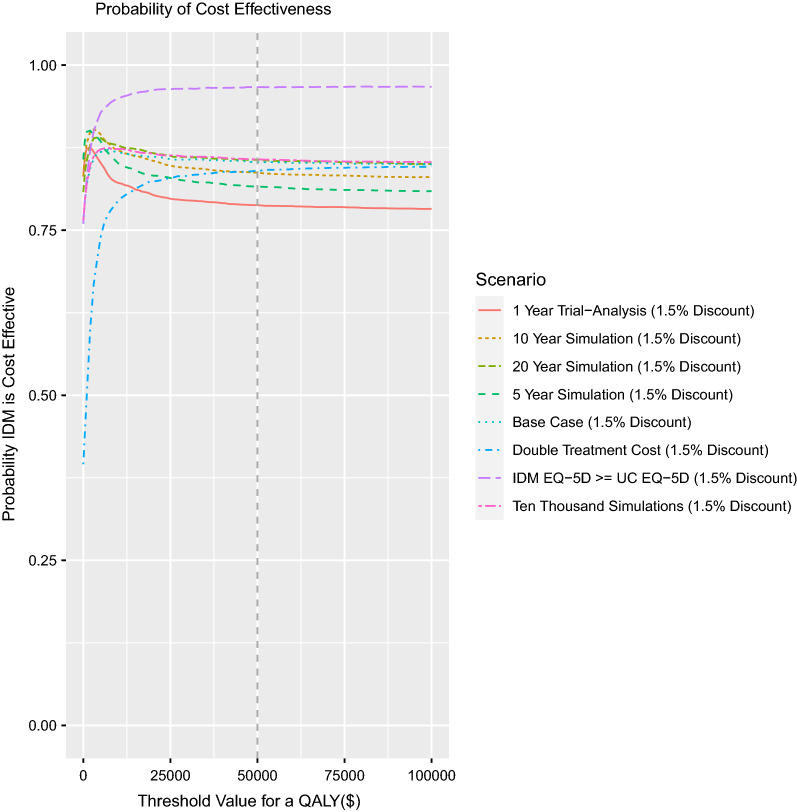
Cost Effectiveness Acceptability Curve. This graph plots the probability that IDM is cost-effective in comparison to UC for a variety of scenarios. The x-axis is a continuous willingness to pay threshold (WTP) in Canadian dollars. The y-axis represents the probability that IDM is cost-effective. The vertical grey-dotted line reflects a WTP of $50,000 per QALY. **1 Year Trial-Analysis (1.5% Discount):** Reflects the one-year trial analysis with a starting age of 68 with a discount rate of 1.5% per annum. **10 Year Simulation (1.5% Discount):** Reflects the model-based analysis if run for 10 years (i.e., 60–70 years of age) with a discount rate of 1.5% per annum. **20 Year Simulation (1.5% Discount):** Reflects the model-based analysis if run for 20 years starting at 68 years (i.e., 68–88 years of age) with a discount rate of 1.5% per annum. **5 Year Simulation (1.5% Discount):** Reflects the model-based analysis if run for 5 years (i.e., 60–65 years of age) with a discount rate of 1.5% per annum. **Base Case (1.5% Discount):** Reflects the base case scenario for the model-based analysis if run for 30 years (i.e., 60–90 years of age) with a discount rate of 1.5% per annum. **Double Treatment Cost (1.5% Discount):** Doubling the cost of all elements associated with the treatment in the IDM group with a discount rate of 1.5% per annum. **IDM EQ-5D >  = UC EQ-5D (1.5% Discount):** Assumes that the utility attributable to the IDM group are always greater than or equal to the utility in the UC group with a discount rate of 1.5% per annum. This reflects the assumption that embedding a CRE within a primary care setting should not negatively impact a patient’s quality of life. **Ten Thousand Simulations (1.5% Discount):** Explores the stability in the estimates of “Base Case (1.5% Discount)” based on 10,000 replications.

The cost-effectiveness acceptability curve of the IDM intervention was calculated based on the probability that the treatment is optimal given different values of WTP for an additional QALY (see Fig. 4). At a commonly used willingness to pay threshold of $50,000/QALY, the trial results demonstrate that the IDM intervention was cost-effective in 78.78% of the replications; IDM was determined to have the highest probability of cost-effectiveness (87.60%) at a WTP of $2,000 per QALY. In the “Base-Case” scenario the IDM intervention was interpreted as cost-effective in 85.30% of the replications; IDM was determined to have the highest probability of cost-effectiveness (87.20%) at a WTP of $7,000 per QALY.

**Fig. 5:**
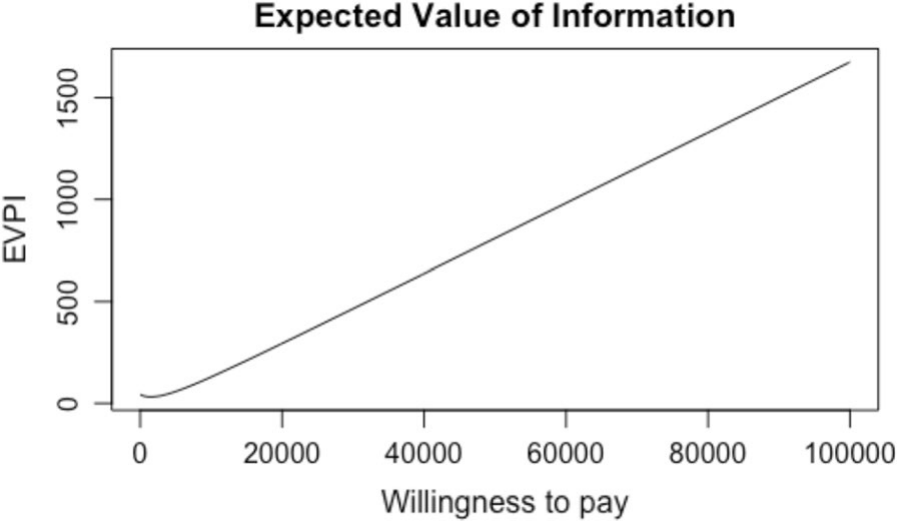
Expected Value of Perfect Information. This graph plots the Expected Value of Perfect Information (EVPI) in dollars ($CAN) (i.e., the y-axis) against a WTP per QALY in dollars (i.e., the x-axis) for the trial-based results.

For the trial-based results the per patient expected value of perfect information per patient (ppEVPI) was calculated to be $809 based on a WTP of $50,000/ QALY (see Fig. 5); in the “Base Case” scenario the ppEVPI was estimated to be $6,266 based on the same WTP. The ppEVPI suggests that any future investment to attempt to reduce the uncertainty of the findings in this study would likely not result in improved decision making if the cost of obtaining that information exceeded the product of the ppEVPI and the potential number of patients affected [33]. To understand the drivers of uncertainty within the “Base Case” cost-effectiveness model, we conducted a robust uncertainty analysis and expanded the number of replications to 10,000; the findings were consistent at 10,000 replications. Specifically, because the ppEVPI for the full model may suggest that future research is warranted, we sought to understand the primary drivers of uncertainty within the model. Further examination of the ppEVPPI suggests that the greatest value for additional research is for perfect information on the EQ-5D differential between the IDM and UC groups (i.e., approximately 75% of the ppEVPI). This finding is also supported by the linear and logistic models that were created to explore uncertainty relative to cost-effectiveness and incremental net-benefit. See supplementary material for additional analysis.

### Scenario analysis

Scenario analyses assumed a WTP of $50,000 per QALY gained (see Fig. 3). IDM remains cost-effective and dominant in comparison to UC under all of the following scenarios: increasing the discount rate in the base case to 5%, running IDM with different time horizons (i.e., one, five and 10 years), assuming that IDM always has more than or equal to the amount of utility in the UC group, and when conducting 10,000 replications of the base case scenario. If the cost of the IDM program were to double, IDM was the optimal treatment in 84.02% of cases (versus 85.30% in the base case) and was cost-effective but not dominant. If we assumed that the utility in the IDM group was always greater than or equal to the UC group, IDM was cost-effective and dominant in 96.66% of replications (versus 85.30% in the base case) and had an ppEVPI of $655 against a WTP of $50,000/QALY. Further, if we assumed that the IDM group had no impact on utility (i.e., the IDM utility estimates were equivalent to UC utility estimates) then assuming a WTP of $0 per QALY (i.e., only interested in the cost of the program) we observe that IDM costs less than UC in 76.94% of replications. We also ran a model with a starting age of 68 years (i.e., reflective of the starting age in the trial analysis) with a time horizon of 20 years and found that IDM was cost-effective and dominant in 85.64% of cases. See supplementary material for full results of scenario analyses.

## Discussion

This study demonstrates, in both our trial-based as well as model-based analyses, that the ‘Best Care’ IDM program for COPD is cost-effective in comparison to the usual standard of care. These important findings highlight the cost-effectiveness of embedding CREs within a primary care setting to support the management of high risk, exacerbation prone patients with COPD. Importantly, this study contributes to the body of literature where, “the (cost) effectiveness of IDM in primary care COPD patients remains unknown” [34, 35]. We acknowledge the limitations of the sample size and the 1-year duration of this study but note that our sample size, duration, and improvements in quality of life (QoL) are consistent with the findings of a recent systematic review that included 26 published IDM international intervention trials with 2,997 patients [36]. We placed a high value on completing the long-term base-case modeling recognizing the significant global economic burden of COPD exacerbations. To address the limitation of extending our analysis beyond one-year we identify that the ‘Best Care’ IDM program is designed as a sustained care relationship with an expectation of sustained impact. We make this assumption given that several core components are demonstrated to have sustained impacts on severe exacerbations, hospitalizations, and QoL including: (1) self-management education with a case manager and a written action plan [26]; (2) annual influenza vaccination [37]; (3) inhaled medication matched to disease severity in frequent exacerbators [38–40]; and (4) our unpublished data demonstrating a sustained improvement in CAT scores over 3-years. We addressed the limitations created by sample size through the uncertainty within our parameter estimates, however we acknowledge that a larger sample size may have improved the precision of our parameter estimates themselves. We also recognize that Markov models may be limited by their requirement to have a definite number of health states and a specific cycle length (i.e., as opposed to modeling health states and time in a continuous manner). With that stated, we have chosen health states and a cycle length that are based on clinical inputs and are consistent with other models of cost-utility and cost-effectiveness analyses that have been used to adjudicate the effectiveness of other COPD interventions as well as other diseases with defined health states [19, 41–45].

Our results are also similar to Bandurska and colleagues (2019) who demonstrated that an integrated care management program for COPD patients had a negative ICER relative to the number of COPD related hospital admissions [34]. For further contextualization we considered published cost-effectiveness analyses done on pulmonary rehabilitation (PR). The comparison of the ‘Best Care’ IDM program with PR programs is appropriate because ‘Best Care’ IDM provides all the elements of a PR program, except for supervised exercise. ‘Best Care’ IDM compares favourably to the cost-effectiveness estimates of other PR interventions, as well as the reasonable cost estimates of COPD PR interventions established by the London Respiratory Network with the London School of Economics (i.e., £2,000- £8,000/ QALY; $3,475- $13,900 CAN/ QALY) that have been adopted within international clinical practice guidelines [2, 41, 46–51]. Specific to the GOLD classification within our study (i.e., GOLD II-IV patients), Atsou and colleagues (2016) modeled a PR program in France and reported a ICER of €17,583 per QALY (i.e., approximately $26,786 CAN) [41]. Another study of PR within a Canadian context reported that PR is cost-effective and demonstrates cost-savings of approximately -$344 per patient, but did not measure aggregate utility and/or QALY [51]. Although we acknowledge that methodological differences in modeling and the variation in health system structures may not permit the direct comparison of interventions, we assert that ‘Best Care’ IDM is consistent, and in some cases dominant, to other cost-effectiveness analyses of similar interventions of IDM.

Pragmatically, it is important to place the findings from our uncertainty analysis in the context of the specific clinical intervention (i.e., specifically as it relates to the utility estimates). Clinically there is an expectation that IDM will not negatively impact utility. In fact, clinical research demonstrates that IDM likely improves utility [36, 52]. Thus, it is reasonable to assume that embedding a CRE within a primary care setting for high risk, exacerbation prone, patients with COPD should not result in decreased utility relative to UC. Therefore, contrary to the uncertainty analysis findings, we assert that additional research seeking to improve the understanding of the QALY differential between IDM and UC may be unnecessary. More pointedly, if we assume that IDM does not have negative implications for utility relative to UC (i.e., IDM utility was greater than or equal to UC utility) we observe that IDM is cost-effective in 96.66% of replications and the ppEVPI is reduced to $655. Furthermore, under the scenario where we assume that IDM utility is equal to UC utility (i.e., there is no benefit/ consequence to utility in the IDM versus UC groups), and we are strictly interested in the scenario where IDM costs less than UC, we observe that IDM costs less than UC in 76.94% of replications. Simply stated, the ppEVPI of the “Base Case” model is likely overstated.

We estimated utility by using a linear regression equation developed to convert individual CAT score measures including: chest tightness, activity, confidence, and energy to EQ-5D [10]. The regression equation used to convert CAT to EQ-5D is reported to sometimes overestimate utility in severe disease states and underestimate utility in healthier patients. [29] Within our analysis, the majority of our study population (i.e., 92% or 132 of the 143 patients enrolled in our study) were classified as GOLD II or GOLD III and were not at the extremes of their health state. To this end, the concern about the estimation of utility is likely mitigated. Further, it is also acknowledged that in the instances where the regression equation is embedded within MCS (i.e., such as in this model) that the algorithm provides estimates of EQ-5D-3L similar to direct measurements and is suitable for cost-effectiveness modeling [29]. We also recognize that the absence of an error term in the regression equation used to estimate utility from CAT is a limitation and minimizes the variability in the estimates for utility. However, because the utilities for both the UC group and IDM group were estimated using the same regression equation, the concern relative to both the estimation of and variation in the utility estimates is of minimal concern for this study as it equally effects both groups [30, 31].

At the initiation of the study, while all patient parameters were generally well matched, it was observed that the mean CAT scores were significantly lower in the UC group in comparison to the IDM group (p < 0.01) (i.e., a lower CAT score results in a higher health utility estimate). While statistically significant, the actual difference was small (i.e., below the threshold for the minimum clinically important difference for the CAT score) and therefore unlikely to meaningfully affect the model. Further to this point, because the UC group had lower CAT scores than the IDM group at study initiation and both the trial-analysis and model-based analyses demonstrate that IDM improves QALY in comparison to UC, the potential QALY improvements of IDM may be understated and generally mitigates this potential limitation. Finally, it is worthwhile to mention that the patients included in this study were almost exclusively Caucasian (i.e., n = 71 in UC, n = 69 in IDM), and from the Ontario/ Canadian universal health care system which may limit the generalizability of the findings.

It should be also noted that this model only considers the direct costs of the IDM program (i.e., related to staffing and physical resources) and assumes that the IDM program can leverage existing resources within a primary care setting at no additional cost, or hinderance, to the primary care provider including indirect expenses (e.g., facilities, utilities, etc.). These expenses may need to be considered by policy makers responsible for the structure of remuneration strategies within primary care. On a year-over-year basis, the IDM program is expected to grow adding additional primary care visits suggesting further consideration may be warranted as it relates to: (1) the capacity of the health system to account for the increased utilization of primary care, and (2) new remuneration strategies for primary care physicians who have “rostered” patients.

Despite the limitations of the study, we contend that our analysis positively contributes to the literature on proactive nonpharmacologic management for high risk, exacerbation prone, patients with COPD in a primary care setting. Specifically, our study demonstrates how CREs can be embedded within a primary care setting to manage these patients in a cost-effective, and dominant, fashion in comparison to the usual standard of care. The extensiveness of our analysis is not an attempt to over extrapolate our results, nor is it an effort to over complicate our model. Rather, we have gone to such lengths to assess the model because of the identified limitations. These efforts reflect our desire to ensure that we are transparently reflecting our results and that the parameters that are driving cost-effectiveness within our model are well understood. What our uncertainty and scenario analyses suggest is that it is very likely that IDM is cost-effective and that the uncertainty in the health utility estimates (i.e., the main drivers of ppEVPI) is moot when we consider that, pragmatically, IDM should not negatively impact health utility values. To this end, because IDM costs less than UC, IDM dominates UC even if there is no observable improvement in health utility. In its’ totality, this study is an important contribution to the literature as it evaluates the cost-effectiveness of a primary care intervention and may have implications for those who may undertake a future weight of evidence (e.g., GRADE) or strength of evidence appraisals of nonpharmacologic management of high risk, exacerbation prone, patients with COPD in a primary care setting.

### Implications for practice & policy

COPD is an ambulatory care sensitive condition and is a leading cause of hospitalization in health systems globally [1, 3]. The 'Best Care' IDM program delivers proactive care that empowers patients in a collaborative self-management model that improves both quality of life and cost-effectiveness. By demonstrating the cost-effectiveness of the ‘Best Care’ IDM program for COPD we confirm that investment in the delivery of evidence based best practices in primary care delivers better patient outcomes at a lower cost (i.e., through a reduction of health service utilization) than the current “as needed” care model in Ontario.

## Conclusion

In conclusion, the ‘Best Care’ IDM COPD intervention has demonstrated cost-effectiveness when deployed in a primary care setting for the management of high risk, exacerbation prone, patients with COPD.

## Supplementary Information

**Additional file 1: Table S1**. Baseline Demographic Information. **Table S2**. Summary of Findings for Scenario Analysis. **Table S5**. Regression Coefficients for CAT to EQ-5D Conversion. Table S6. EQ-5D Estimates that Inform the Full Model. **Figure S1**. Scenario Analysis Cost Effectiveness Acceptability Curve. **Figure S2**. Residual Plot Informed by the Logistic Model. **Figure S3**. Q-Q Plots of Costs & Effects informed by the Logistic Model

## Data Availability

The datasets generated and/or analysed during the current study are not publicly available due to the requirement to keep individual patient health information confidential but are available from Dr. Licskai on reasonable request.

## Abbreviations

COPD: Chronic obstructive pulmonary disease
ED: Emergency department
GOLD: Global initiative for chronic obstructive pulmonary disease
$${FEV}_{1}$$: Forced expiratory volume in one second
IDM: Integrated disease management
PCIC: Primary Care Innovation Collaborative
UC: Usual care
MCS: Monte-Carlo simulation
CRE: Certified respiratory educator
CAT: COPD Assessment Test
ATS: American Thoracic Society
ERS: European Thoracic Society
ER: Emergency room
CADTH: Canadian Agency for Drugs and Technologies in Health
ARGI: Asthma Research Group Windsor-Essex County Inc.
MPD: Medical Program Director
PC: Program Coordinator
QALY: Quality-adjusted life year
ppEVPI: Per patient expected value of perfect information
BCEA: Bayesian cost-effectiveness analysis
Base Case: 30-Year model-based analysis starting at 60 years of age
WTP: Willingness to pay threshold
ICER: Incremental Cost-Effectiveness Ratio
ICUR: Incremental Cost-Utility Ratio
QoL: Quality of Life
PR: Pulmonary Rehabilitation
CAN: Canadian Dollars ($)
