# Supplementary material for: Cost-effectiveness of integrated disease management for high risk, exacerbation prone, patients with chronic obstructive pulmonary disease in a primary care setting
Source: Cost Eff Resour Alloc. 2022 Aug 12;20:39. doi: 10.1186/s12962-022-00377-w (PMC9373353; doi:10.1186/s12962-022-00377-w)
Supplement: Supplementary file 1 — Additional file 1: Table S1. Baseline Demographic Information. Table S2. Summary of Findings for Scenario Analysis. Table S5. Regression Coefficients for CAT to EQ-5D Conversion. Table S6. EQ-5D Estimates that Inform the Full Model. Figure S1. Scenario Analysis Cost Effectiveness Acceptability Curve. Figure S2. Residual Plot Informed by the Logistic Model. Figure S3. Q-Q Plots of Costs & Effects informed by the Logistic Model [file 12962_2022_377_MOESM1_ESM.docx]

**Supplementary Material**

**Baseline Demographic Information**

Table 1 provides a baseline demographic information that was collected at study initiation (i.e., month 0).

Table 1- Baseline Demographic Information

|  | **Usual Care Group** (n=73) | **IDM Group** (n=70) |
| --- | --- | --- |
| Female* | 49.3% (n=36) | 60% (n=42) |
| Male* | 50.7% (n=37) | 40% (n=28) |
| Age (mean; median)* | 67.7; 68 | 69.5; 70 |
| % Caucasian* | 97.3% (n=71) | 98.6% (n=69) |
| GOLD A (n) | 1 | 2 |
| GOLD B (n) | 9 | 8 |
| GOLD C (n) | 3 | 0 |
| GOLD D (n) | 60 | 60 |
| GOLD II (n; mean CAT score; median CAT score)* | 44; 19.75; 20 | 47; 22.51; 21 |
| GOLD III (n; mean CAT score; median CAT score)* | 22; 19.55; 19 | 19; 22,47; 23 |
| GOLD IV (n; mean CAT score; median CAT score)* | 7; 17.43; 17 | 4; 25; 27 |
| n = number of participants  *measured at baseline (i.e., study initiation/ month 0) | | |

**Scenario Analysis**

The following is a summary of the results of each individual scenario analysis that was conducted to contrast IDM vs. UC (see Table 2 & Figure 1). When we assume that IDM EQ-5D = UC EQ-5D, we assume a WTP of $0. We assume a WTP of $0 because we have made the assumption that we would not expected IDM to improve QALY relative to UC. To this end, we are only interested in the scenario that IDM costs less than UC. The adoption of a WTP of $0 places all the emphasis on cost differential. To this end, when WTP is $0, an incremental net-benefit greater than $0 signals that IDM costs less than UC. All other scenarios are presented as described in the table.

Table 2- Summary of Findings for Scenario Analysis

| **Scenario** | **ICUR** | **Cost** | **QALYs** | **Life Years** | **WTP (000)** | **INB** | **P(CE)** | **ppEVPI** |
| --- | --- | --- | --- | --- | --- | --- | --- | --- |
| Trial-Analysis*^ | $2,620 | -$267 | 0.10 | 0 | $50 | $5,360 | 0.7878 | $809 |
| Base Case* | -$2,294 | -$3,973 | 1.73 | 0.244 | $50 | $90,576 | 0.8530 | $6,266 |
| Base Case (5% Discount) | -$2,497 | -$3,071 | 1.23 | 0.139 | $50 | $64,562 | 0.8508 | $4,398 |
| Double Treatment Cost* | $1,099 | $1,936 | 1.76 | 0.245 | $50 | $86,110 | 0.8402 | $6,792 |
| IDM Utility = UC Utility* | -$23,658 | -$4,067 | 0.17 | 0.254 | $0 | $12,663 | 0.7694** | $1,474 |
| IDM Utility $\geq$ UC Utility* | -$1,760 | -$3,849 | 2.19 | 0.259 | $50 | $113,213 | 0.9666 | $655 |
| One Year Simulation* | -$2,403 | -$253 | 0.11 | 0 | $50 | $5,256 | 0.7926 | $804 |
| 5 Year Simulation* | -$2,744 | -$1,378 | 0.50 | 0.01 | $50 | $26,488 | 0.816 | $2,891 |
| 10 Year Simulation* | -$2,827 | -$2,577 | 0.91 | 0.04 | $50 | $48,153 | 0.837 | $3,969 |
| Ten Thousand Simulations* | -$2,307 | -$4,067 | 1.76 | 0.252 | $50 | $92,227 | 0.8572 | $6,087 |
| 20 Year Simulation^ | -$2,392 | -$3,795 | 1.59 | 0.276 | $50 | $83,128 | 0.8564 | $5,569 |
| Cost = Incremental Cost (IDV vs. UC); ICUR = Incremental Cost Utility Ratio; Incremental Net Benefit (INB) = Differential in QALY * WTP per QALY – Differential in Cost; Life Years = Incremental Life Years (IDM vs. UC); ppEVPI = Per Patient Expected Value of Perfect Information at specified WTP; QALYs = Incremental Quality Adjusted Life Years (IDM vs. UC); P(CE) = Probability of Cost Effectiveness at specified WTP; WTP = Willingness to Pay Threshold per QALY in thousands of dollars ($CAN).  *Assume a discount rate of 1.5% per annum, a starting age of 60 years and a time horizon of 30 years unless stated otherwise.  **Calculates the probability that the cost of IDM is less than the cost of UC (i.e., assuming at WTP = $0).  ^Assumes a starting age of 68 years with a 1-year time horizon unless stated otherwise and a discount rate of 1.5% | | | | | | | | |

Figure 1- Scenario Analysis Cost Effectiveness Acceptability Curve


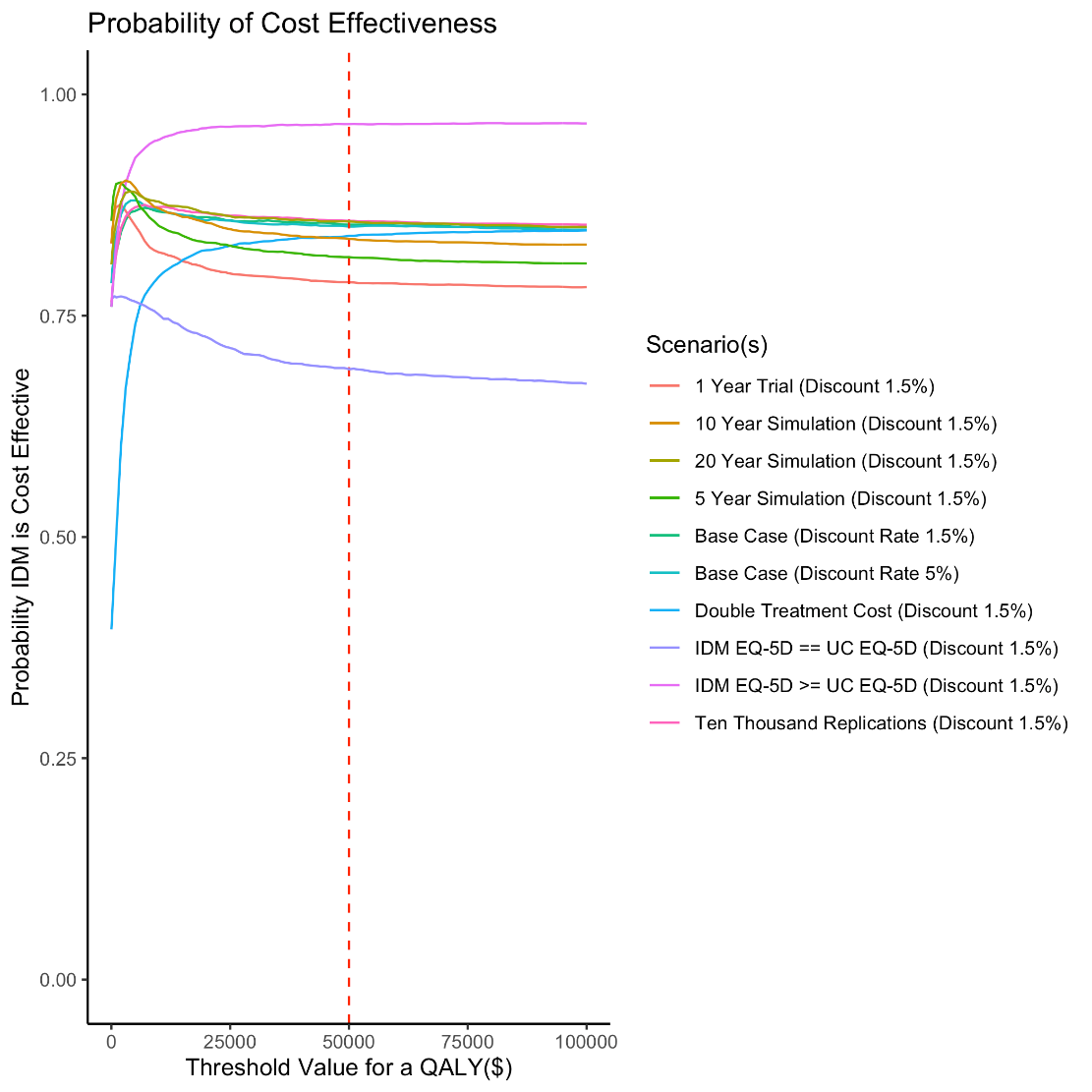


**Uncertainty Analysis**

An analysis was undertaken to understand which parameters influenced the cost-effectiveness of the IDM for high risk, exacerbation prone, patients with COPD. The analysis was expanded to 10,000 replications within a Monte Carlo Simulation. The results of this scenario reported that IDM was cost-effective, and dominant, in comparison to the UC group and had a probability of cost-effectiveness of 85.72% against a willingness to pay threshold of $50,000 (CAN) per QALY. The expected value of perfect information per patient (ppEVPI) was $6,087. Importantly, the probability that the IDM program was cost-effective in 10,000 replications did not differ materially from the probability of cost-effectiveness based on 5,000 unique replications (i.e., 85.72% vs. 85.30%) suggesting that there is stability within the analysis at 5,000 replications.

***Description of Analysis***

In an effort to identify those inputs whose variance have the most influence upon output variation we used multivariate regression techniques with the inputs specifying the set of regressors (explanatory variables) and one of two outputs serving as the response variable. Specifically the two outputs of interest were: setting the response variable to be the value of incremental net-benefit (INB) calculated for each m-tuple of inputs defining a simulation case, or for the purposes of our alternative output, transforming INB into a logical denoted NB (i.e., INB>0).  This yields a matrix X for the regressors of dimension nr (r connoting replications) by ni, where nr is the number of replications in the simulation (10,000 herein) and ni is the number of inputs included as regressors.  To define the response variable (Y), we use a column vector labelled Y with dimension nr x 1 (capturing either INB or NB).  Anticipating that EQ-5D weights would emerge as especially influential and noting that at issue is not the utility weight for both IDM and UC for any one COPD condition but rather the difference in EQ-5D weight (IDM versus UC) for each of the COPD stages, we replaced the 6 EQ-5D inputs with three Delta inputs, capturing the COPD conditioned matched differences.  The resulting X matrix was then transformed taking each input a replacing it with its z-score counterpart: (x-xbar)/s.x where x is the input value, xbar is the inputs mean, and s.x is the inputs standard deviation.   The purpose of the latter step is to obviate nuisance scale (e.g., what units in input was measured in) scale effects.

***Linear and Logistic Models***

With the resulting X matrix and Y vector in hand, we performed multivariate regression (Y~X) using conventional linear regression fitting procedures when INB was the response (Y) variable and using generalized linear model fitting procedures (logistic) when the output was NB (Y).  Under these approaches the absolute value of the fitted coefficients for each input convey their relative contribution to output variation^1,2^.  The two regressions (linear and logistic) revealed consistent results, ranking the aforementioned DeltaEQ5D inputs as explaining the most output variation (INB or NB in case of logistic).  It was noteworthy that a model restricted to include just these three inputs was able to explain 85% of the output variation (based on R^2 statistic).  We acknowledge that the mathematics of the CEA model would not suggest that the outputs could be self-evidently explained by a simple linear combination of the inputs (which our Y~X side approach attempts to do), but highlight the impressive R^2 value as suggesting that approximating the CEA model as a linear combination explains a good deal of the variation.  One way to allow for nonlinearities is to transform each of the nr values for an input into their ranks (akin to a Spearman approach to exploring correlation).  We did this for the linear model (i.e., not the logistic model) and found that our findings of the three DeltaEQ-5D inputs being most influential, was unchanged.  The same three inputs achieved top ranks when we conducted a stepwise forward regression starting from the null model --- they were the first inputs entered into the null model by the stepwise procedure, being chosen in the following order.

***Expected Value of Perfect Partial Information***

To assess which variables informed cost effectiveness, we employed a logistic model without interaction terms. ‘Cost-effectiveness’ (i.e., INB > 0) was the dependent variable and the standardized model parameters were the independent variables; variables associated with the cost of an: urgent care visit, emergency room visit, hospitalization, and treatment, were aggregated to be individual values for each group to avoid collinearity (e.g., Cost of Hospitalization = Hospital Stay + Laboratory and Diagnostic Test + Transportation). The analysis was completed using a stepwise Akaike Information Criteria (AIC) selection in the backwards directions. The model with the smallest AIC value included 31 parameters including: Relative Risk of Death (GOLD II & III), Hazard Ratio of Hospitalized Patients (GOLD III & GOLD IV), Probability of Transition in the UC group (GOLD II to III, & III to IV), Probability of Exacerbation in the UC group (GOLD III & IV), Probability of Hospitalization in the UC group (GOLD II-IV), Frequency of Hospitalization in the UC group (GOLD II-IV), Probability of Transition in the IDM group (GOLD II to III, & III to IV), Probability of Exacerbation in the IDM group (GOLD II-IV), Probability of Hospitalization in the UC group (GOLD II-IV), Frequency of an Urgent Care visit in the IDM group (GOLD III & IV), Frequency of an Emergency Room visit in the IDM group (GOLD IV), Frequency of Hospitalization in the IDM group (GOLD II-IV), the Cost of Treatment, and the Difference in EQ-5D between the IDM group and the UC group (GOLD II-IV). Collinearity amongst the identified parameters within the model was checked against the variance inflation factor (VIF). The majority of parameters reported VIF scores that were greater than but close to 1, with the exception of the DeltaEQ5DII (i.e., the difference in IDM vs UC EQ-5D estimates for GOLD II) and DeltaEQ5DIII (i.e., the difference in IDM vs UC EQ-5D estimates for GOLD III) which reported VIF scores that were greater than 3 but less than 4.

To assess the specific variables of interest within the 31 variables that were identified by the logistic model to inform cost-effectiveness, an expected value of perfect partial information (EVPPI) analysis for selected parameters was undertaken. The EVPPI of the 31 variables resulted in a value of approximately $5,313, or approximately 87% of the ppEVPI of the full model with 10,000 replications (i.e., ppEVPI = $6,087). To ensure this model was normally distributed against the cost-effectiveness analysis, we examined the residuals (see Figure 1) and Q-Q plots (see Figure 2). Through exploring a residual plot of the logistic model against the cost-effectiveness analysis we are generally satisfied that there is no discernable trend and that the costs and effects (i.e., QALYs) are normally distributed. We can confirm this quasi-qualitative interpretation through an exploration of the Q-Q plots for costs and effects. For the effects we observe a near perfect normal distribution. The Q-Q plot as it relates to cost is roughly normally distributed within +/- 2 standard deviations (SD), however we see strong tails deviating away from the normal distribution beyond +/- 3 SD. This is not that surprising within the healthcare context as there are a select number of patients who use substantially more healthcare resources than other patients. To this end, we are not overly concerned about the distribution of the residuals and conclude that the logistic model is an appropriate fit to inform our analysis of EVPPI.

Figure 2- Residual Plot Informed by the Logistic Model


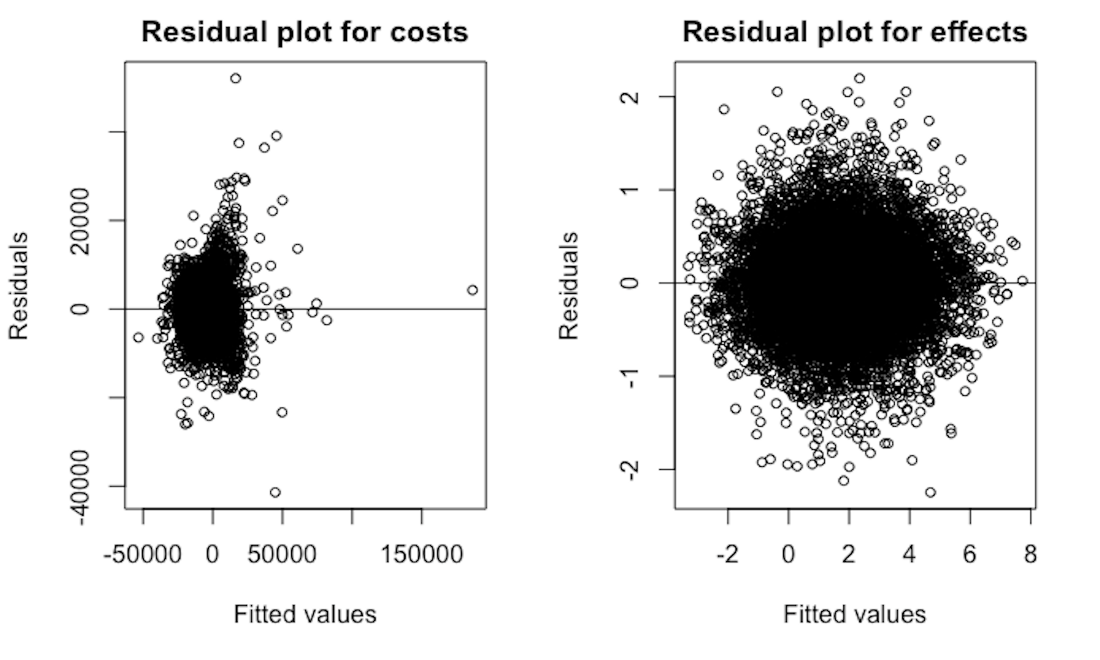


Figure 3- Q-Q Plots of Costs & Effects informed by the Logistic Model


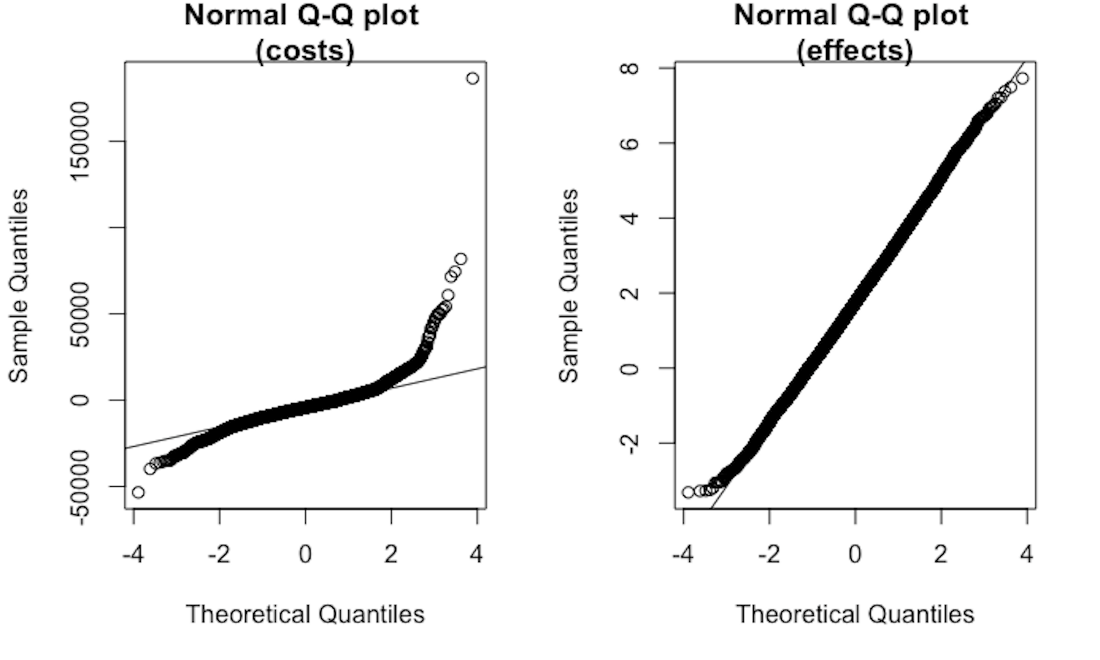


Further analysis of specific groups of parameters (e.g. parameters related to QALY, etc.) revealed that the EVPPI for DeltaEQ5D for the group of GOLD II, III & IV parameters, was equivalent to $4,726 or approximately 89% of the EVPPI for all 31 variables. Against a willingness to pay threshold of $50,000 per QALY the EVPPI analysis identified that, relative to the EVPPI, the DeltaEQ5DII, DeltaEQ5DIII, and the Frequency of Hospitalization for GOLD IV in the IDM group were individually informative and had EVPPI greater than $0. This supports the findings from the analysis conducted by the linear and logistic models.

Moreover, this emphasizes that uncertainty in cost-effectiveness is largely driven by EQ-5D estimates. This is an important finding given that the cost of IDM treatment was observed to be less than that of the UC treatment. To this end, even if the IDM treatment had no effect on EQ-5D (i.e., assuming that IDM and UC have the same effect on EQ-5D), the IDM treatment would still be the more desirable treatment in comparison to UC given IDM has the potential to result in cost savings. Specifically, if we were to assume that there was no utility difference between IDM and UC (i.e., IDM EQ-5D is equal to UC EQ-5D) then the decision would likely be most interested in the potential for cost savings (i.e., does IDM cost less than UC), as opposed to interested in the potential to improve utility. To this end, when assuming that the potential for cost savings is the variable of interest (i.e., at WTP of $0 per QALY) we observe that IDM costs less than UC in 76.94% of simulations. Further, if we assume that the IDM must always be greater than or equal to the amount of utility in the UC group, we find that IDM is cost effective in 96.66% of simulations (i.e., 5,000 replications).

**Utility Estimates**

As described in the full text of the manuscript, utility estimates (i.e., EQ-5D) were derived using linear regression of specific CAT score measures (i.e., chest tightness, activity, confidence, and energy) from the original patient population in the original randomized control trial (see Table 5). In lieu of creating a theoretical distribution around each specific utility estimate characterized by each GOLD classification (respective of IDM and UC), we use resampling with replacement as a mechanism to estimate utility. Because utility estimates were derived from a linear regression used to convert CAT scores (i.e., model ‘M3t_OLS’ as per Hoyle et al. (2016)) resampling with replacement was perceived to be the favorable method of estimating utility within the model. It was not possible to impose theoretical distributions on the CAT scores because of a high degree of covariance between individual CAT scores^3^. To permit reproducibility of the results, Table 6 summarizes utility values used in the model.

Table 5- Regression Coefficients for CAT to EQ-5D Conversion

| **Parameter** | **Coefficient** | **Source** |
| --- | --- | --- |
| Regression Coefficient- Constant | 0.978 | ^3^ |
| Regression Coefficient- CAT Chest Tightness | -0.0257 | ^3^ |
| Regression Coefficient- CAT Activities | -0.0330 | ^3^ |
| Regression Coefficient- CAT Confidence | -0.0426 | ^3^ |
| Regression Coefficient- CAT Energy | -0.0188 | ^3^ |

Table 6- EQ-5D Estimates that Inform the Full Model

| **EQ-5D Estimates** | | | | | |
| --- | --- | --- | --- | --- | --- |
| **GOLD II- UC** | **GOLD II- IDM** | **GOLD III- UC** | **GOLD III- IDM** | **GOLD IV- UC** | **GOLD IV- IDM** |
| 0.641 | 0.712 | 0.703 | 0.797 | 0.576 | 0.773 |
| 0.762 | 0.882 | 0.641 | 0.745 | 0.585 | 0.540 |
| 0.377 | 0.660 | 0.497 | 0.858 | 0.726 | 0.636 |
| 0.922 | 0.870 | 0.571 | 0.755 | 0.544 | 0.801 |
| 0.747 | 0.799 | 0.778 | 0.733 | 0.818 | 0.771 |
| 0.764 | 0.635 | 0.629 | 0.743 | 0.889 | 0.797 |
| 0.561 | 0.717 | 0.472 | 0.686 | 0.823 |  |
| 0.764 | 0.907 | 0.729 | 0.732 |  |  |
| 0.710 | 0.820 | 0.606 | 0.752 |  |  |
| 0.820 | 0.823 | 0.618 | 0.959 |  |  |
| 0.755 | 0.978 | 0.650 | 0.978 |  |  |
| 0.785 | 0.645 | 0.513 | 0.907 |  |  |
| 0.696 | 0.803 | 0.641 | 0.952 |  |  |
| 0.844 | 0.934 | 0.901 | 0.856 |  |  |
| 0.849 | 0.893 | 0.753 | 0.764 |  |  |
| 0.700 | 0.753 | 0.615 | 0.915 |  |  |
| 0.703 | 0.658 | 0.838 | 0.549 |  |  |
| 0.731 | 0.723 | 0.585 | 0.693 |  |  |
| 0.495 | 0.634 | 0.738 | 0.729 |  |  |
| 0.557 | 0.863 | 0.959 | 0.856 |  |  |
| 0.729 | 0.893 | 0.686 |  |  |  |
| 0.660 | 0.959 | 0.767 |  |  |  |
| 0.743 | 0.729 | 0.660 |  |  |  |
| 0.856 | 0.896 | 0.842 |  |  |  |
| 0.825 | 0.934 | 0.766 |  |  |  |
| 0.735 | 0.940 | 0.701 |  |  |  |
| 0.780 | 0.806 |  |  |  |  |
| 0.781 | 0.889 |  |  |  |  |
| 0.745 | 0.804 |  |  |  |  |
| 0.712 | 0.830 |  |  |  |  |
| 0.488 | 0.566 |  |  |  |  |
| 0.818 | 0.915 |  |  |  |  |
| 0.547 | 0.660 |  |  |  |  |
| 0.566 | 0.884 |  |  |  |  |
| 0.767 | 0.908 |  |  |  |  |
| 0.616 | 0.825 |  |  |  |  |
| 0.693 | 0.856 |  |  |  |  |
| 0.643 | 0.875 |  |  |  |  |
| 0.823 | 0.797 |  |  |  |  |
| 0.712 | 0.780 |  |  |  |  |
|  | 0.870 |  |  |  |  |
|  | 0.844 |  |  |  |  |
|  | 0.922 |  |  |  |  |
|  | 0.842 |  |  |  |  |

**References:**

1. Morgan MG, Henrion M, Small M. *Uncertainty: A Guide to Dealing with Uncertainty in Quantitative Risk and Policy Analysis*. Cambridge university press; 1990.

2. Iman RL, Helton JC. An Investigation of Uncertainty and Sensitivity Analysis Techniques for Computer Models. *Risk Analysis*. 1988;8(1):71-90. doi:10.1111/j.1539-6924.1988.tb01155.x

3. Hoyle CK, Tabberer M, Brooks J. Mapping the COPD Assessment Test onto EQ-5D. *Value in Health*. 2016;19(4):469-477. doi:10.1016/j.jval.2016.01.005
